# Hyaluronic acid injection to coronal sulcus of the penis for the treatment of premature ejaculation: a retrospective observational study

**DOI:** 10.1186/s12894-023-01214-9

**Published:** 2023-04-01

**Authors:** Kewei Chen, Qing Li, Tao Xu, Xiaowei Zhang

**Affiliations:** Urology, Peking University People’s Hospital, Beijing, China

**Keywords:** Hyaluronic acid, Premature ejaculation, Injectable technique

## Abstract

**Background:**

Hyaluronic acid (HA) injection has become a burgeoning method to treat premature ejaculation (PE) due to its high biocompatibility and structural properties.

**Purpose:**

In this study, we proposed a modified technique: injecting hyaluronic acid around coronal sulcus to treat PE, aiming to decrease the complications of hyaluronic acid injection in penis while achieving similar effects.

**Method:**

A total of 85 patients who had HA injection from January 2018 to December 2019 were analyzed retrospectively in our study. 31 patients received injection at glans penis and 54 patients received injection around coronal sulcus. Intravaginal ejaculation latency time (IELT) was mainly measured to estimate the efficacy and the severity of complications was assessed between two groups.

**Results:**

The mean IELT was 123.0 ± 37.28 s of all patients, 124.7 ± 39.01 s of patients injecting at glans penis and 121.9 ± 36.58 s of patients injecting around coronal sulcus. IELT of all patients increased to 482.1 ± 121.7 s at 1 month, 331.2 ± 81.2 s at 3 month and 280 ± 80.4 s at 6 month. In the group of injecting at glans penis, the incidence of complications is 25.8% and it is 1.9% in the group of injecting around coronal sulcus. No severe complication was reported in both groups.

**Conclusion:**

The modified technique of injecting around coronal sulcus decreases complications and it has the potential to become a new injectable technique for treating premature ejaculation.

## Introduction

Premature ejaculation (PE) is a common male sexual dysfunction, affecting approximately 5% of men in the general community [1]. Although PE does not damage the lifespan, the patients’ psychological health, even their self-esteem and relationship with partners is in potential impact zone. With the rising safety of soft tissue filler technologies, more and more men are undergoing surgery to treat PE. As a novel procedure, hyaluronic acid (HA) injection is less dangerous than traditional procedures. Despite HA is not prone to produce complications because of its high biocompatibility and structural properties, it is not an absolutely safe soft-tissue augmentation filler due to the residue of bacterial fermentation and the special anatomy of the glans penis [2]. To reduce surgical complications, several injection techniques have been proposed. Early on, linear threading was established to make the injection procedure simple and effective, however there are several complications, such as mucosal tearing, hemorrhage, and leaking through the needle site [3]. Subsequently the multiple puncture approach, developed by Abdallah et al. results in a more even distribution of HA gel while causing less soreness [4]. However, surgical methods for reliving PE reported by previous literature are similar to glans augmentation with HA gel rather than a simple approach to treat PE [5, 6]. To some extent, the relief of PE is a side effect of glans penis augmentation with HA gel in most previous literature [5, 7, 8]. For these reasons, a modified injectable technique is proposed: injecting HA around coronal sulcus with a multi puncture injection. We performed this study to verify the hypothesis that injecting around coronal sulcus has a beneficial effect on reducing complications.

## Materials and methods

### Patients

A total of 85 PE patients received HA therapy from January 2018 to December 2019 were analyzed retrospectively in our study. All patients had lifelong or acquired PE and desired to relieve the symptoms of PE through surgical intervention with no medicine. The International Society of Sexual Medicine’s (ISSM) definition of PE was approved. It proposed inclusion of an objective, quantifiable time to ejaculation, which is referred to as the intravaginal ejaculatory latency time (IELT). The IELT is defined as the time from vaginal penetration to ejaculation. Lifelong PE is characterized by an IELT of < 1 min since first intercourse, whereas IELT of < 3 min at any point in a man’s life is considered to be acquired PE [1].

With reference to previous indications and contraindications, all of our patients meet inclusion and exclusion criteria as follows: (1) Lifelong or acquired PE; (2) Aged above 18 and below 70; (3) No alcoholism or drugs abuse; (4) No history of surgery related to extend IELT or taking Sildenafil or other similar drugs in 6 months [4, 6, 9]. Other medication of psychotherapy, surgeries or drugs of treating PE are not allowed during our study. All patients signed an informed consent form. This study was approved by the local ethics committee and informed consent was obtained by all subjects when they were enrolled.

### Methods

**Fig. 1:**
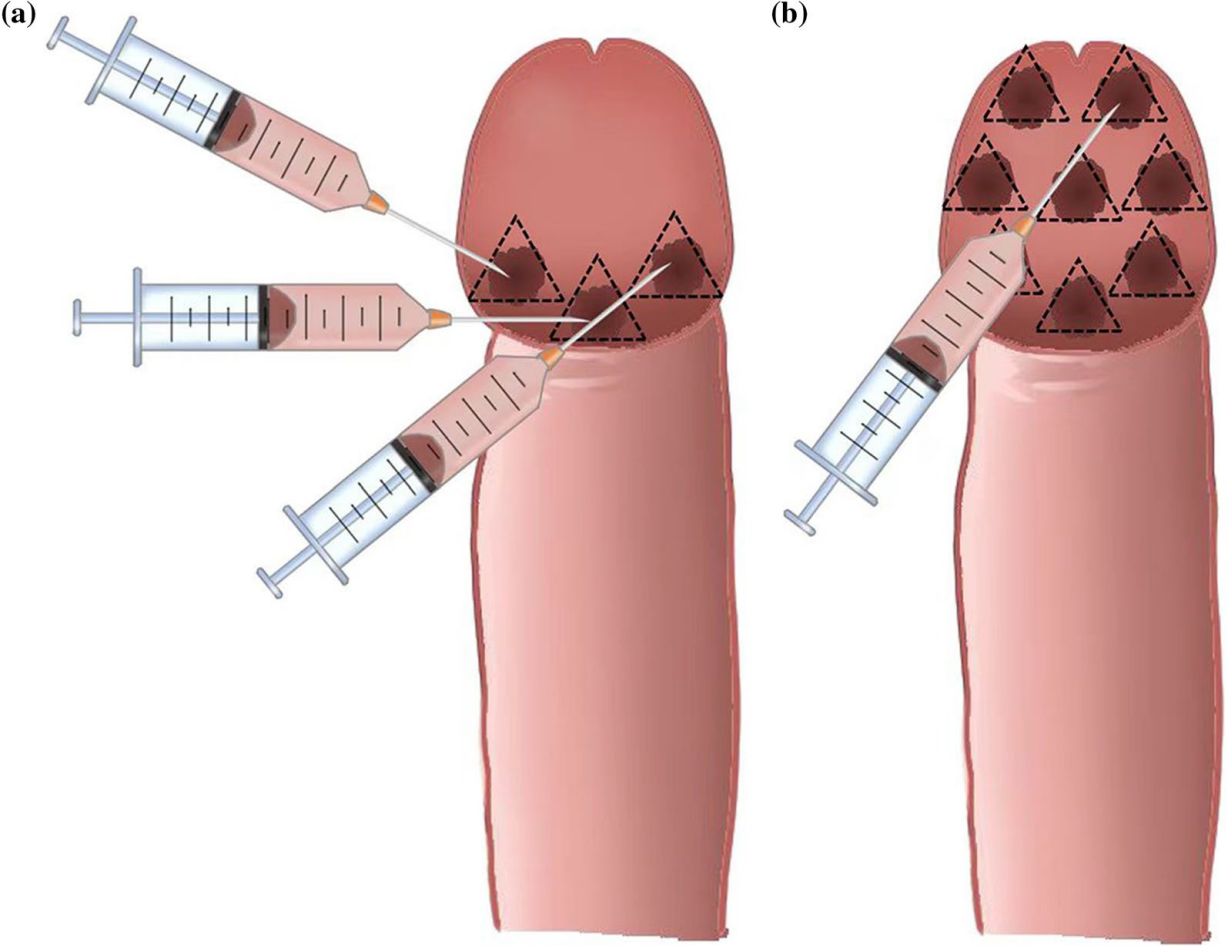
**a** The diagrammatic presentation of coronary sulcus injection shows that hyaluronic acid is only located in the coronary sulcus area after injection, and the glans penis will not be significantly enlarged. **b** The schematic diagram of Fan technique shows that the hyaluronic acid nodule fills the entire glans glans after regional injections

Each procedure was accomplished by an experienced surgeon. Every patient was required to maintain supine position during the period of injection procedure. The patient’s perineum area was sterilized twice by 0.25% iodophor to prevent infection. The penis and scrotum were sterilized at first, then the penis was wrapped with sterile gauze and the foreskin was pushed backward to expose the urethral orifice. Then the urethral orifice, glans penis and coronal sulcus were sterilized, other areas including the anus were sterilized finally. The sterilized area is upward to the umbilicus, bilateral to the posterior axillary line, and downward to the upper third of the thigh. The antibiotics was not routinely used to prevent infection before surgery. Local anesthesia was subsequently performed by lidocaine gel 25 mg distributed on the glans, especially on the coronal sulcus and glans penis. The modified injection technique is described below. We indwelled a 27G needle to inject hyaluronic acid gel (Perfectfill, Gallop, Shandong, China) around the coronal sulcus, then adopted multipuncture injectable technique, whose punctures were surrounded with coronal sulcus and deposited 0.1–0.2 ml HA gel in each puncture. The depth and the amount of HA of each injection, a total of 8–12 injection sites divided coronal sulcus evenly, were the same. The injection sequence was from the ventral side to the dorsal side of the penis, and the area around the urethral opening was not injected (Fig. 1a). The Fan technique is as follows. The injection needle was inserted through the tunnel between the penile superficial fascia and Buck’s fascia at the proximal one-third from the tip of the glans to the coronal sulcus and it was rotated continuously along the glans to administer HA gel evenly (Fig. 1b). After the injection, correct the surface undulation by injecting HA using a 30G needle. If observing nodules after injection, suitable massage for the glans penis, especially the area around the coronal sulcus was indispensable. To standardize confounding factors, all interventions were completed by the same experienced urology surgeon and the type, quantity and cross-link of HA were same.

The evaluation was done by measuring IELT and premature ejaculation profile (PEP). The patients were required to restart sexual intercourse 1 week after the intervention. The partner held the timer to count the time from insertion into the vagina to the start of ejaculation as IELT. When patients received follow-up, several IELTs were filled in the questionnaire because of several timer measurements, the median IELT of all was taken as the effective IELT. Sexual intercourse was observed a total of 6 months. In addition, patients were asked to complete PEP questionnaire to evaluate the degree of improvement of PE. Moreover, the patient's satisfaction was evaluated by 4 levels: dissatisfied, a little satisfied, satisfied, very satisfied, which were scored as 0, 1, 2, and 3.

### Statistical analysis

Data analysis were conducted with SPSS (version 25.0; SPSS, Chicago, IL, USA) for Windows. Mean standard deviation/range, minimum and maximum were presented for quantitative variables. We use mean ± standard deviation (SD) to express demographic information for all subjects and data (including IELT, PEP and satisfaction) at baseline (before operation) and 1-month, 3-month and 6-month follow-up visit for all subjects. Mann–Whitney and Wilcoxon signed ranks tests were used to compare the differences between two groups. Chi-square test was used to compare differences between groups. *P* < 0.05 was considered to be statistically significant.

## Result

**Fig. 2:**
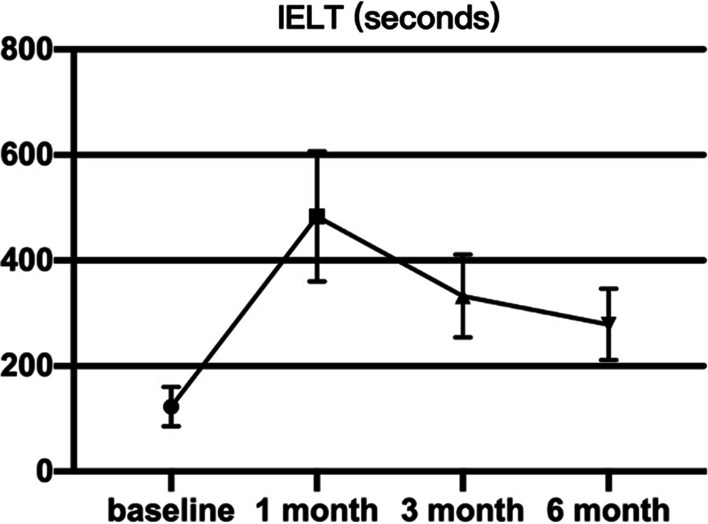
Evaluation of IELT of all patients who received HA injecting by both techniques. IELT illustrated as mean and min-maximum range at baseline and after 1, 3 and 6 months of HA injection. All of them are compared to baseline and show significant difference from baseline

A total of 85 patients received HA injection were analyzed retrospectively in our study. 31 patients received HA injection into glans penis (Group 1) and 54 patients received HA injection around the coronal sulcus (Group 2). The demographic data of all patients are shown in Table 1. The mean age of the patients was 34.30 ± 6.72 years old and the mean age of their partners was 31.6 ± 4.2 years old. The mean IELT before intervention was 124.7 ± 39.01 s of Group 1 and 121.9 ± 36.58 s of Group 2. IELT was mainly measured to evaluate the effect of HA therapy (Table 2). IELT of all patients increased from to 482.1 ± 121.7 s at 1 month, 331.2 ± 81.2 s at 3 month and 280 ± 80.4 s at 6 month (Fig. 2). IELT of patients injecting at glans penis increased to 475.9 ± 130.9 s at 1 month, 325.8 ± 71.26 s at 3 month and 282.2 ± 62.38 s at 6 month. IELT of patients injecting around coronary sulcus increased to 487.9 ± 119.9 s at 1 month, 336.6 ± 82.77 s at 3 month and 276.8 ± 71.02 s at 6 month. The IELT of both groups at 1, 3, 6 month was statistically significant compared with the baseline (*P* < 0.001). Group 1 and Group 2 did not show significant statistical difference in prolonging the latency of ejaculation (*P* > 0.05).

**Table 1:** Demographic data of all patients

Type of injecting technique	Injecting at glans penis	Injecting around coronal sulcus
Patients’ age (years)	32.2 ± 5.0	33.5 ± 5.1
Partners’ age (years)	31.6 ± 4.2	31.8 ± 4.7
Marriage duration (years)	6.4 ± 3.2	6.9 ± 4.1
IELT (seconds)	124.7 ± 39.01	121.9 ± 36.58

The table shows the demographic information of the cases included in the study, all displayed in the mean ± standard deviation

**Table 2:** Comparison of 1 month, 3 month and 6 month versus baseline by both techniques

Type of injecting technique	Injecting at glans penis			Injecting around coronal sulcus		
Period of follow-up	1 month	3 month	6 month	1 month	3 month	6 month
IELT (seconds)	475.9 ± 130.9	325.8 ± 71.26	282.2 ± 62.38	487.9 ± 119.9	336.6 ± 82.77	276.8 ± 71.02
Median difference versus baseline (seconds)	346	222	161	347	200	157.5
*P* value (vs. baseline)	< 0.001*	< 0.001*	< 0.001*	< 0.001*	< 0.001*	< 0.001*

The table shows the IELT at 1, 3, and 6 months after the operation of the cases included in the study, and is shown by the mean ± standard deviation and median respectively

*IELT* Intravaginal ejaculation latency time

*Wilcoxon matched-pairs signed rank test was used to compare scores of 1, 3, 6 month follow-up to baseline

The PEP scores is shown in Table 3. After HA gel injection, the PEP scores of patients in both Group 1 and Group 2 were improved and the scores of 1, 3, and 6 month follow-up showed significant statistical differences from the baseline (*P* < 0.001). The PEP scores in Group 1 and Group 2 at the same follow-up period were also compared, but there is no significant statistical difference. Similarly, patients’ satisfaction with sexual intercourse improved after the intervention. The satisfaction scores of Group 1 is 0.672 ± 0.581, 3.16 ± 0.820, 2.81 ± 0.833 and 2.613 ± 0.919 at baseline, 1, 3, and 6 months respectively. The satisfaction scores of Group 2 is 0.604 ± 0.556, 3.13 ± 0.825, 2.96 ± 0.823 and 2.83 ± 0.863 at baseline, 1, 3, and 6 months respectively. There was no statistically significant difference between the two groups in the same follow-up period regardless of satisfaction (*P* > 0.05). Table 4 shows satisfaction scores and statistical differences between two groups.

**Table 3:** PEP scores of 1 month, 3 month, 6 month by both techniques

Type of injecting technique	Injecting at glans penis				Injecting around coronal sulcus			
Period of follow-up	Baseline	1 month	3 month	6 month	Baseline	1 month	3 month	6 month
Scores of question 1	0.968 ± 0.795	3.32 ± 0.791	3.13 ± 0.718	2.16 ± 1.13	1.00 ± 0.847	3.19 ± 0.646	3.056 ± 0.811	2.59 ± 1.04
Scores of question 2	0.807 ± 0.654	3.032 ± 0.658	3.13 ± 0.763	2.36 ± 1.05	0.870 ± 0.728	3.09 ± 0.680	3.04 ± 0.800	2.24 ± 1.10
Scores of question 3	0.742 ± 0.631	3.32 ± 0.599	3.29 ± 0.824	2.26 ± 1.13	0.815 ± 0.646	3.11 ± 0.744	3.02 ± 0.789	2.39 ± 1.09
Scores of question4	1.23 ± 1.02	2.968 ± 0.605	3.36 ± 0.755	2.32 ± 1.11	1.17 ± 0.947	2.815 ± 0.646	3.11 ± 0.817	2.35 ± 1.05
P* value (Group1vs. Group2)	Question 1	Baseline 0.9336		1 month 0.2302		3 month 0.7543	6 month 0.0955	
	Question 2	Baseline 0.7957		1 month 0.6834		3 month 0.6495	6 month 0.6900	
	Question 3	Baseline 0.6455		1 month 0.2355		3 month 0.1005	6 month 0.6751	
	Question 4	Baseline 0.8735		1 month 0.2784		3 month 0.1817	6 month 0.9847	

In 1, 3, and 6 months of follow-up, all of PEP scores of Group 1 and Group 2 showed statistical differences (*P* < 0.001) compared with the baseline by Mann–Whitney and Wilcoxon signed ranks tests. All data is displayed in the mean ± standard deviation

*Wilcoxon matched-pairs signed rank test was used to compare scores of 1, 3, 6 month follow-up to baseline

**Table 4:** Satisfaction scores of 1 month, 3 month, 6 month by both techniques

Type of injecting technique	Injecting at glans penis				Injecting around coronal sulcus			
Period of follow-up	Baseline	1 month	3 month	6 month	Baseline	1 month	3 month	6 month
Score	0.672 ± 0.581	3.16 ± 0.820	2.81 ± 0.833	2.613 ± 0.919	0.604 ± 0.556	3.13 ± 0.825	2.96 ± 0.823	2.83 ± 0.863
P* value (vs. baseline)	–	< 0.001	< 0.001	< 0.001	–	< 0.001	< 0.001	< 0.001
P* value (Group1 vs. Group2)	Baseline 0.9659	1 month 0.8842	3 month 0.4293	6 month 0.2699				

*Wilcoxon matched-pairs signed rank test was used to compare scores of 1, 3, 6 month follow-up to baseline. Mann–Whitney and Wilcoxon signed ranks tests was used to compare two groups. All data is displayed in the mean ± standard deviation

### Complications

**Fig. 3:**
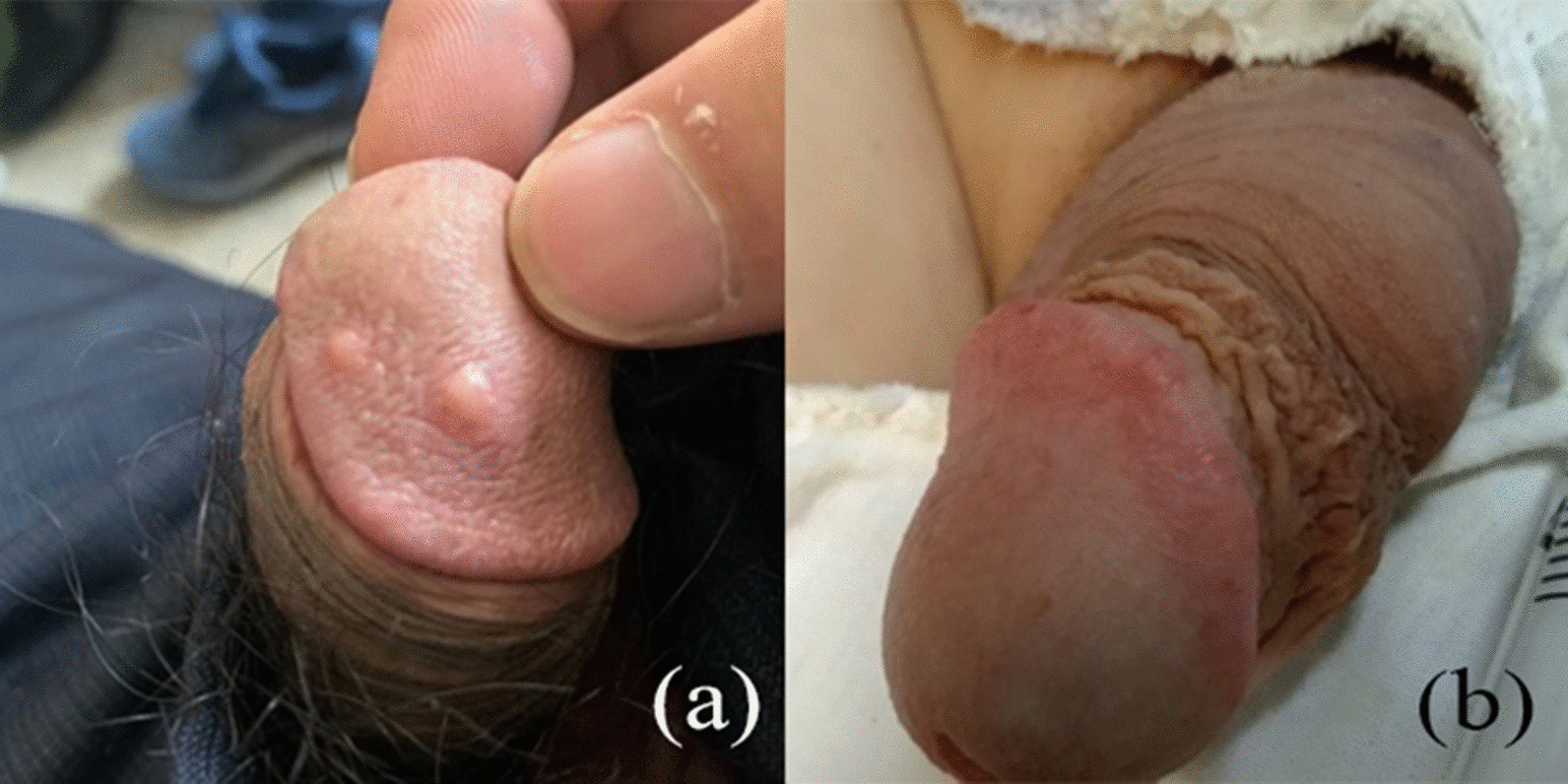
Subcutaneous HA nodule at the penis. **a** The front view of a patients developed 2 subcutaneous HA nodules at the glans penis on the 6th day post intervention. **b** The front view of a patients developed fluctuation around coronal sulcus on the 5th day post intervention. HA gel is distributed more uniform by injecting around coronal sulcus. The HA nodules is revealed by nodes at the glans penis while fluctuation around coronal sulcus

A total of 9 patients (10.6%) with complications were reported in 85 patients after HA injection. The complication rates in the group 1 and group 2 are 25.8% and 1.9%, respectively (Table 5).The most frequent complications are subcutaneous HA nodules (Fig. 3), regional vascular embolism and skin necrosis. The most common complication was HA nodules, whose incident rate was 19.4% in Group 1 and 1.9% in Group 2. The rate of vascular embolism and skin necrosis were namely 6.5% and 3.2% in Group 1. There is no vascular embolism and skin necrosis in Group 2. The complications appeared most in 1-month after intervention. Even though various complications were observed, most of them were resolved after moderate massage and pressure bandaging in 1 month. HA still cannot be evenly distributed in a few nodules after massage. At this time, we reduce the size of nodules by injecting hyaluronidase, which usually takes about 3 days to take effect, and promote the redistribution of HA by pressure bandaging. During the follow-up, we found that the only person with skin necrosis was because the patient had diabetes and secondary infection. We took the way of removing necrotic tissue and injecting hyaluronidase to solve it. Besides, there were no systemic effects or multiple organ dysfunction throughout the whole follow-up period.

**Table 5:** Complications of all patients

Type of injecting technique	Injecting at glans penis	Injecting around coronal sulcus
HA nodules	6	1
Vascular embolism	2	0
Skin necrosis	1	0
Total number of patients	8	1
Incidence	25.8%*	1.9%*

The table shows the type and number of side effects during follow-up

*P = 0.0011 by Chi test

## Discussion

Several drug therapies, including local anesthetics, selective serotonin reuptake inhibitors and tramadol, have been used to treat PE. Dorsal neurectomy is one of the options for the treatment of PE. As a polysaccharide existing in the intercellular matrix of dermal layers of the skin of all species, it is attracting people's attention [2, 7, 10]. HA has both the high biocompatibility making foreign body responses less and the capacity create hydrated polymers with high viscosity, which makes it the perfect soft tissue filler [2, 7, 10, 11]. Thanks to mentioned properties, HA has been used as a safe soft-tissue filler for decades in various plastic surgery operations [7, 12]. With the progressive application of HA in the field of andrology, it has shown a certain effect in the treatment of PE, which prolongs the latency of vaginal ejaculation [4–6, 11, 13, 14]. According to the current evidence, HA PE treatment improved IELT and was safe and well tolerated, with reported adverse events ranging from 0 to 30% in all research. Localized soreness, protrusion, lump and numbness of the scrotum were the primary adverse effects, which resolved spontaneously in 2 weeks [15].

The specific mechanism of HA injection in treating PE is still unclear, but a possible hypothesis has been proposed. The reduction in glans sensation in the glans penis, the creation of barriers between the stimulating factor and receptor may all contribute to the effect of injecting HA gel into the glans penis to raise IELT. [5, 16]. In addition, the increase in confidence of placebo effect helps. Abdallah et al. reported the improvement in IELT in 49 patients after enlargement of the glans penis with HA from a mean of 127.2 to 462.6 and 319.2 s after 1 and 3 months respectively [4]. Analogously, a randomized controlled cross-over study by Littara et al. indicates IELT improves from a mean of 88.34–293.14 s in 110 patients after 6 months [6]. Compared to previous studies, the extent of IELT extension was similar in our research.

**Fig. 4:**
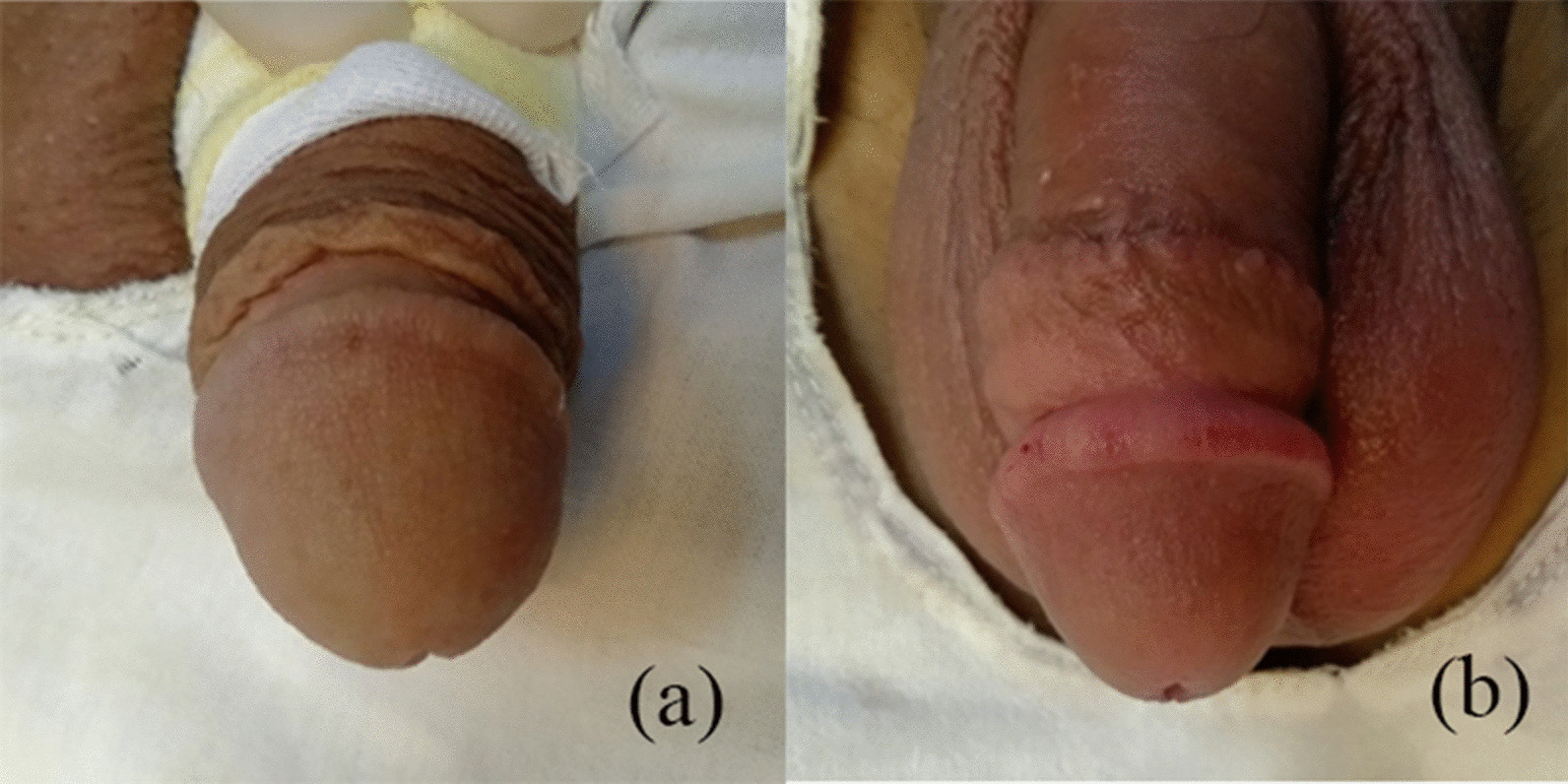
Comparison of the shape of the penis before and after intervention. **a** The shape of the glans penis of the patient before intervention. **b** The shape of the glans penis of the patient on the 30th day post intervention. It was only slightly enlarged at the coronal sulcus and the overall appearance was more esthetics

**Fig. 5:**
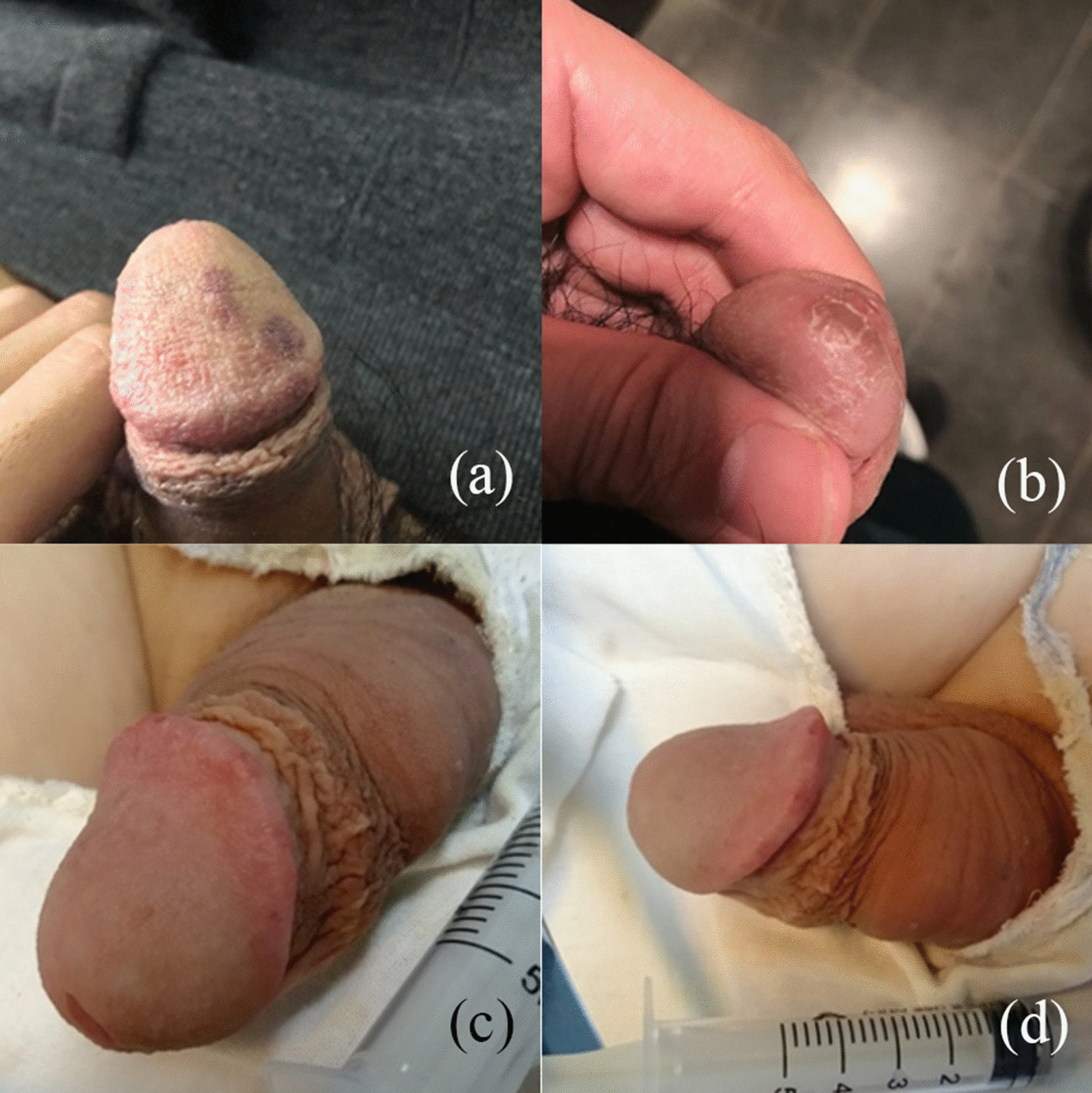
Regional vascular embolism and nodule at glans penis and no severe complications by the new technique. **a** The patient had slight regional vascular embolism manifested by dark red skin one day after the injection. **b** The patient had late onset nodule manifested by skin ulceration followed on the 10th day post intervention. **c** The front view of a patient’s shape of the glans after injection. **d** The side view of this patient

As an affordable, nonsurgical alternative for correcting contour defects and soft tissue augmentation [17], HA injecting has its own unique advantages of simple operation and ability to combine with other surgeries. Although the incidence of complications is low, several common complications are still observed. Abdallh et al. reported 7 patients having complications with multiple puncture technique and 7 patients having complications with Fan technique and the percentage was 26.9% and 30.4% respectively [4]. However, Littara et al. analyzed 171 patients who received HA injection, of whom 0 had adverse reactions [6]. In addition to above studies, Amr Alahwany et al. reported 6 patients in 30 patients (20%) with adverse effects after 1 week of HA injection [11]. What’s more, Dae Yul Yang et al. reported 3 patients (9.1%) with complications in 33 patients [9]. Overall, the incidence is between 0 and 30.4%. Among these adverse effects, HA nodules, local discomfort, ecchymosis, papule formation and glans numbness are most common [3, 10, 15]. The possible causes of side effects include the low purity of HA gel, excessive gel injection, the incorrect layer of location, or injection into the blood vessels [18]. In our study, similar types of complications as above were observed. As the most common complications, HA nodules are mainly caused by uneven distribution of HA gel. Although the redistribution of HA can be speeded up by massaging the nodules, if the injection is too fast or the nodules are too large, the nodules will not disappear easily even after 1–2 months of massage. Surgical resection and hyaluronidase are effective treatments for large nodules. Compared to Fan technique, one advantage of our injecting technique decreases HA nodules after HA injection. Compared to glans penis surface, the stratum corneum of coronal sulcus is thicker, which means more space for HA and less likely to be swelling and form HA nodules. Some literatures may not consider the HA nodules formed after injection as a complication, but this does bring troubles and aesthetic dissatisfaction to a number of patients. Our simplified injection method significantly reduces the occurrence of hyaluronic acid nodules (Fig. 4). In our study, the percentage of complication of group 1 and group 2 are 25.8% and 1.9% respectively. When HA is injected around coronal sulcus, there are no severe complications observed (Fig. 5).

Regional vascular embolism was the most severe complication. If vascular embolism is severe, skin necrosis will occur further. The main reason of vascular embolism is that HA is injected into the vessels in the corpus cavernosum of penis. If the injection is too superficial, the above-mentioned HA nodules and fluctuations will occur, but if it is too deep, there is a risk of injecting HA into the blood vessel and thus vascular embolism [13]. For a patient with skin necrosis in this study, removing necrotic tissue and injecting hyaluronidase was adopted. Antibiotics are also used to fight infection. The patient recovered the basic appearance of the penis 2 months later. HA injecting around coronal sulcus can effectively avoid the risk of vascular embolism, because the coronal sulcus is farther away from the blood vessel.

On the basis of less adverse reactions, the effect of treating PE by coronal sulcus injection is as promising as the previously reported injection method. Because of the more complicated and scattered distribution of nerves on the glans, surgical procedures on this area of the penis may result in some serious adverse effects. Therefore, incisions in the glans should be limited, and if needed, should be made as distally and little as possible [19, 20]. We noticed that anatomy does not acquire importance in previous studies and the principle of avoiding the distal end is not taken into account. Based on the above clinical anatomy, we believe that HA injection at the margin of coronal sulcus is safer than that at glans.

In addition to the advantages mentioned above, our injection method has the following strongpoints. First of all, the difficulty of injection technique is the challenge of using HA injection to treat PE. Combined with multipuncture technique, it decreases HA nodules or fluctuation on the glans by HA injecting around coronal sulcus, which actually simplifies the injecting technique. As a result, inexperienced doctors can quickly master this technique, which is beneficial to verify efficacy. Second, as some patients do not have any obstacles to the appearance of the penis, such as concealed penis and small penis syndrome, their purpose of coming to see a doctor is simply to improve the symptoms of PE caused by hypersensitivity of dorsal nerve branches or psychological factors. One advantage of our surgical method is that it meets the demands of patients who only want to treat PE rather than enlargement of the glans. At last, multiple injections of HA will increase the incidence of complications [13], but our technology will not produce severe complications, which makes multiple injections possible. It has the hope of becoming a feasible standard procedure for the treatment of PE.

However, this study still has some limitations. First, we did not divide the glans and the coronal sulcus into several areas for injection separately to compare the aesthetic effect and the incidence of complications. In addition, large enough patients and long-term follow-up are necessary to determine further efficacy. And we did not have a control group considering the effect of injecting HA at glans penis have been verified in lots of literatures.

## Conclusion

This study illustrates that aiming to treat premature ejaculation by injecting HA around the coronal sulcus, the incidence of complications will be reduced without changing the efficacy. In addition, this injection technology solves the problem of complicated and difficult to quantify injection technology in the past. Therefore, we hope that through the improvement of injection methods, complications of HA injection treatment would be significantly reduced.

## Data Availability

The data that support the findings of this study are available from the corresponding author upon reasonable request.
